# Supplementary material for: Clinical characteristics of re-positive COVID-19 patients in Huangshi, China: A retrospective cohort study
Source: PLoS One. 2020 Nov 4;15(11):e0241896. doi: 10.1371/journal.pone.0241896 (PMC7641455; doi:10.1371/journal.pone.0241896)
Supplement: S1 Table — (DOC) [file pone.0241896.s001.doc]

**S1 Table: Univariate analysis of laboratory indices of patients with and without RP at discharge.**

|  | **Non-RP group(n=362)** | **RP group(n=21)** | **HR (95% CI)** | **P** |
| --- | --- | --- | --- | --- |
| White blood cell count, ×10^9^ per L | 5.41(1.87 4.44-6.31) | 5.35(2.95, 4.47-7.42) | 1.08(0.84,1.40) | 0.5259 |
| Lymphocyte count, ×10^9^per L | 1.53(0.70, 1.17-1.87) | 1.62(0.44, 1.36-1.80) | 1.57(0.74,3.33) | 0.2398 |
| Neutrophil count, ×10^9^per L | 0.50(0.29, 0.36-0.65) | 0.50(0.37, 0.40-0.77) | 1.59(0.31,8.08) | 0.5729 |
| Platelet count, ×10^9^per L | 235.00(100.00, 192.00-292.00) | 229.00(97.00, 175.00-272.00) | 1.00(0.99,1.00) | 0.7838 |
| AST, U/L | 24.00(15.00, 19.00-34.00) | 23.00(19.00, 18.00-37.00) | 0.98(0.94,1.01) | 0.1914 |
| ALT, U/L | 31.50(38.00, 20.00-58.00) | 22.00(47.00, 12.00-59.00) | 0.98(0.97,1.00) | 0.0754 |
| Lactate dehydrogenase, U/L | 176.00(61.00, 155.00-216.00) | 164.00(62.00, 136.50-198.50) | 0.99(0.98,1.00) | 0.0681 |
| Troponin I, ng/ml | 0.01(0.01, 0.01-0.02) | 0.03(0.05, 0.01-0.06) | NA | NA |
| B-type brain natriuretic peptide, pg/ml | 20.10(35.40, 10.00-45.40) | 48.34(175.91, 25.84-201.75) | 1.00(1.00,1.01) | 0.0755 |
| C-reactive protein, mg/L | 1.69(4.18, 0.82-5.00) | 1.00(2.19, 0.46-2.65) | 0.83(0.66,1.05) | 0.1178 |
| ESR, mm/h | 36.00(49.00, 20.50-69.50) | 55.00(82.00, 13.00-95.00) | 1.01(0.99,1.04) | 0.3477 |
| D-Dimer, μg/mL | 0.21(0.39, 0.11-0.50) | 0.20(0.48, 0.08-0.56) | 0.69(0.17,2.84) | 0.6077 |
| Activated partial thromboplastin time, s | 33.40(5.60, 31.10-36.70) | 32.90(5.20, 30.50-35.70) | 0.98(0.90,1.07) | 0.6282 |

Data are median (IOR, 1st-3rd) or n (%). P values were calculated as Wald tests.
