# Supplementary material for: Clinical characteristics of re-positive COVID-19 patients in Huangshi, China: A retrospective cohort study
Source: PLoS One. 2020 Nov 4;15(11):e0241896. doi: 10.1371/journal.pone.0241896 (PMC7641455; doi:10.1371/journal.pone.0241896)
Supplement: S2 Table — (DOC) [file pone.0241896.s002.doc]

**S2 Table: Laboratory indices of RP patients at re-admission**

| **RP patients in re-admission** |  |
| --- | --- |
| **No. of patients** | 23 |
| **Laboratory findings** |  |
| White blood cell count, ×10^9^ per L | 5.63(1.62, 5.41-7.03) |
| Lymphocyte count, ×10^9^per L | 1.67(0.36, 1.45-1.81) |
| Neutrophil count, ×10^9^per L | 3.43(1.46, 3.01-4.47) |
| Platelet count, ×10^9^per L | 232.00(52.00, 211.00-263.00) |
| AST, U/L | 22.50(13.50, 17.50-31.00) |
| ALT, U/L | 26.50(27.00, 13.00-40.00) |
| Lactate dehydrogenase, U/L | 167.00(44.30, 143.00-187.30) |
| Troponin I, ng/ml | 0.052(0.051, 0.033-0.084) |
| B-type brain natriuretic peptide, pg/ml | 47.10(24.76, 42.95-67.71) |
| C-reactive protein, mg/L | 1.21(0.59, 0.92-1.51) |
| ESR, mm/h | 11.00(7.75, 5.25-13.00) |

Data are median (IQR, 1st-3rd)
