# Supplementary material for: Clinical characteristics of re-positive COVID-19 patients in Huangshi, China: A retrospective cohort study
Source: PLoS One. 2020 Nov 4;15(11):e0241896. doi: 10.1371/journal.pone.0241896 (PMC7641455; doi:10.1371/journal.pone.0241896)
Supplement: S3 Table — (DOC) [file pone.0241896.s003.doc]

**S3 Table : Antibody results of 15 RP-patients.**

| **Antibody results** | **IgG** | **IgM** |
| --- | --- | --- |
| **RP Patient No.1** | **+** |  |
| **RP Patient No.2** | **+** |  |
| **RP Patient No.3** | **+** |  |
| **RP Patient No.4** | **+** |  |
| **RP Patient No.5** | **+** | **+** |
| **RP Patient No.6** | **+** |  |
| **RP Patient No.7** | **+** |  |
| **RP Patient No.8** | **+** |  |
| **RP Patient No.9** | ± |  |
| **RP Patient No.10** | **+** |  |
| **RP Patient No.11** | **+** | ± |
| **RP Patient No.13** | **+** |  |
| **RP Patient No.14** | **+** |  |
| **RP Patient No.15** | **+** |  |
| **RP Patient No.18** | **+** |  |
