# Supplementary figures and images for: Clinical characteristics of re-positive COVID-19 patients in Huangshi, China: A retrospective cohort study
Source: PLoS One. 2020 Nov 4;15(11):e0241896. doi: 10.1371/journal.pone.0241896 (PMC7641455; doi:10.1371/journal.pone.0241896)

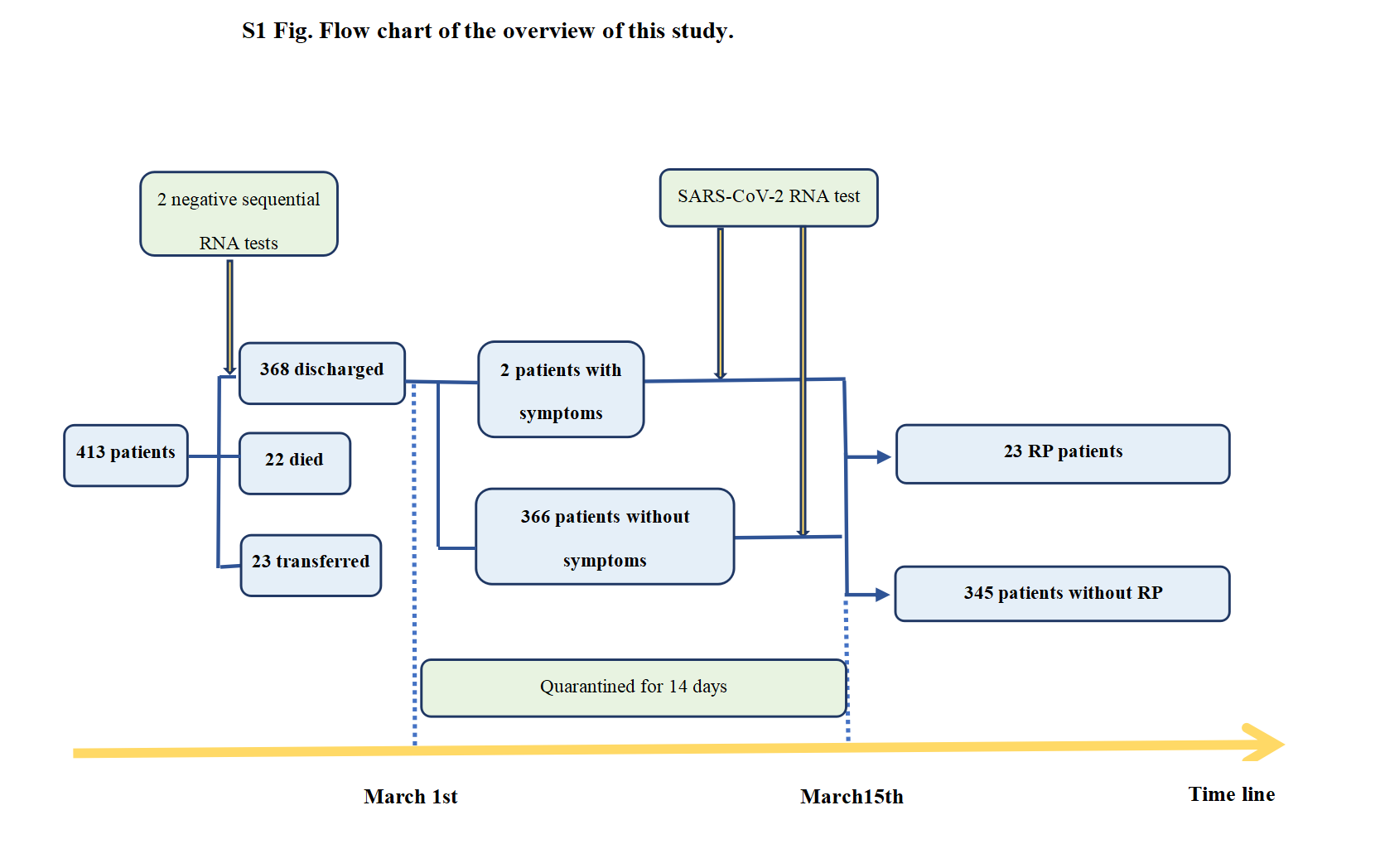

Supplement: S1 Fig — (TIF) [file pone.0241896.s004.tif]
