# Supplementary material for: Clinical characteristics of re-positive COVID-19 patients in Huangshi, China: A retrospective cohort study
Source: PLoS One. 2020 Nov 4;15(11):e0241896. doi: 10.1371/journal.pone.0241896 (PMC7641455; doi:10.1371/journal.pone.0241896)
Supplement: S1 File — (DOC) [file pone.0241896.s005.doc]

# Clinical characteristics of Re-positive patients with COVID-19 in Huangshi, China: a retrospective cohort study

# Case Report Form

Patient initials □□□□

DATE Year Month

**Admission profile**

1. **Basic information**

Admission date： Y M D

Medical record number：

Date of birth： Y M

Age:

Gender：□1.Male □2.Female

clinical diagnosis：

**Exposure to source of transmission within past 14 days**： □1.Recently visited Wuhan □2. Had contact with Wuhan residents □3.Had contact with the Confirmed COVID19 patients□4.Family gathering history □5.None

**Incubation period**：

Diagnosed Date：

**Initial signs and symptoms**：□1.Fever □2.Cough □3.Sputum production

□4.Chest tightness □5.Fatigue □6.Hyperhidrosis □7.Insomnia

□8.Dizzy □9.Nasal congestion □10.Chills □11.Pharynx itching □12.ache

1. **Past history**

Coronary Heart Disease □1.No □2.Yes

Hypertension □1.No □2.Yes

Chronic Respiratory diseases □1.No □2.Yes

Chronic renal disease □1.No □2.Yes

Diabetes □1.No □2.Yes

Cancer □1.No □2.Yes

Leukemia □1.No □2.Yes

Liver disease £1.No □2.Yes

Others：1.

2.

1. **Physical examination (on admission)**

Temperature ._℃ Heart rate

Respiratory rate 4.Height cm Body weight Kg

Breathing rhythm Pulmonary rales

£1.Regular □2.Irregular □1.No □2.Yes

**laboratory indices on admission**

| **Measurement items** | **Measured value** | **Unit** |
| --- | --- | --- |
| **Routine blood test** |  |  |
| RBC |  | 10^12^/L |
| Hb |  | g/L |
| WBC |  | 10^9^/L |
| PLT |  | 10^9^/L |
| LYM |  | 10^9^/L |
| Ne |  | 10^9^/L |
| **Routine urine test** |  |  |
| RBC |  | 个/HP |
| WBC |  | 个/HP |
| Protein |  | G/L |
| **Biochemistry Test** |  |  |
| ALT |  | Iu/L |
| AST |  | Iu/L |
| BIL |  | μmol/L |
| BUN |  | mmol/L |
| Cr |  | μmol/L |
| **Others** |  |  |
| Tropolin I |  | ng/ml |
| BNP |  | pg/ml |
| CRP |  | mg/L |
| ESR |  | mm/H |
| D-D2 |  | μg/L |
| APTT |  | s |
|  |  |  |

**Inpatient data**

1. **Treatment in hospital**

£1.Arbidol £2.Lopinavir /Ritonavir

□3.Ribavirin □4.Ganciclovir

□5.Arbidol + Lopinavir/Ritonavir □6.α-interferon nebulization

□7.Inhaled corticosteroid □8.Systemic corticosteroid

1. **Discharge outcome：** □1.Dead □2.Discharge □3.Transferred

Discharge date：

Length of stay：

Total duration of disease：

Peak temperature during hospitalization：

Duration of fever during hospitalization：

1. **Severity of illness**：□1.mild □2.moderate □3.severe □4.critical

**laboratory indices on discharge**

| **Measurement items** | **Measured value** | **Unit** |
| --- | --- | --- |
| **Routine blood test** |  |  |
| RBC |  | 10^12^/L |
| Hb |  | g/L |
| WBC |  | 10^9^/L |
| PLT |  | 10^9^/L |
| LYM |  | 10^9^/L |
| Ne |  | 10^9^/L |
| **Routine urine test** |  |  |
| RBC |  | 个/HP |
| WBC |  | 个/HP |
| Protein |  | G/L |
| **Biochemistry Test** |  |  |
| ALT |  | Iu/L |
| AST |  | Iu/L |
| BIL |  | μmol/L |
| BUN |  | mmol/L |
| Cr |  | μmol/L |
| **Others** |  |  |
| Tropolin I |  | ng/ml |
| BNP |  | pg/ml |
| CRP |  | mg/L |
| ESR |  | mm/H |
| D-D2 |  | μg/L |
| APTT |  | s |
|  |  |  |

**Signature of collector：**

**Date of collection：**

**Auditor statement**

I have reviewed all the contents and data in this case report form, and confirm that the information records are true and accurate, and the items are completely filled in, which meets the requirements of the trial protocol.

**Signature of auditor————————**

**Audit date————————**
