# Supplementary material for: Clinical characteristics of re-positive COVID-19 patients in Huangshi, China: A retrospective cohort study
Source: PLoS One. 2020 Nov 4;15(11):e0241896. doi: 10.1371/journal.pone.0241896 (PMC7641455; doi:10.1371/journal.pone.0241896)
Supplement: Attachment — Submitted filename: Response to Reviewers.docx [file pone.0241896.s007.docx]

**Dear editor:**

Thanks a lot for having reviewed our manuscript.   Now we have revised the manuscript according to the reviewers’ comments. Most of the revisions are in the manuscript. Some explanations regarding the revisions of our manuscript are as follows. During the revison, we found that a few patients were transfered between two hospitals and excluded them in new manuscript. Thank you very much for your comments. Your questions have played a very important role in improving this research.

Our manuscript was re-edited by Editage (www.editage.com) on your recommendation.

**Answers to Editor:**

(1) **The authors need to discuss whether re-positive patients after recovery was due to second infection or they were still contiguous? An antibody result will help to address this question.**

Reply：Thank you very much for your suggestion. We reviewed data and looked up the relevant information about the antibody results. Because our patients were followed-up earlier (Before March 1), only 15 re-positive patients in all 23 re-positive patients were tested for antibodies. All these 15 patients showed positive antibody results (2 patients with IgM positive, 15 patients with IgG positive; detailed information can be found in S3 Table). There is one article named “Humoral Immune Response to SARS-CoV-2 in Iceland” published in the New England Journal recently. Their results indicate that antiviral antibodies against SARS-CoV-2 did not decline within 4 months after diagnosis [1]. In rhesus macaques，researchers found out a relationship between a humoral immune response to SARS-CoV-2 infection and protection against reinfection by this virus [2]. To our knowledge, there was no article reported re-infection of SARS-Cov-2 in such a short time in the presence of positive antibody. Therefore, we speculate that the re-positive patients in this study are less likely to be caused by the second infection. According to your advice, we have added the antibody results and the discussion of re-infection in the manuscript.

**(2) The term of "re-positive recovering (RPR) patients" is awkward; do you mean re-positive patients after recovery or discharge?**

Reply：In our original vision, we wanted to describe the clinical characteristics of asymptomatic re-positive patient , so we defined such a description. However, we had to admit that this description is really awkward. After repeated discussions in our group， we replaced the term “re-positive recovering (RPR) patients” with “re-positive (RP) patients ” in new manuscript and two symptomatic re-positive patients were also included in study .

**(3) Formats of all numbers in abstract, contents and tables need to change, for example, changing "P=0·0015" into "P=0.0015"**

Reply：We have made improvements in accordance with your requirements.

(4) **in many tables, it was stated that median (IQR) was used; however, i am doubtful for that. Did you mean S.D. since there was only one digit presented? For IQRs, 1st and 3rd IQR are generally used. Please change all the values into median (IQR, 1st and 3rd) in all of the tables.**

Reply：Thanks for your advice. We have changed our values in tables as you mentioned.

**(5) in the discussion, authors stated that "... they might have a reduced anti-viral immune response." I disagree on it. These patients most likely were due to residual viral loads, second likely re-infection.**

Reply：Thank you for your question. One study from Hong Kong, researchers could not amplify active viruses from samples of re-positive patients [3]. Thus, it is possible that the presence of re-positive patients was likely to be caused by broken virus fragments or dead viruses that are not completely cleared in the body. And talking about re-infection, we considered the possibility was relatively low. Firstly，15 of 23 re-positive patients underwent antibody test, and all of them showed positive results. There was no article reported re-infection of SARS-Cov-2 in such a short time in the presence of positive antibody. Secondly, all patients were very strictly quarantined after discharge from hospital within 14 days. They had no chance to exposure to the source of the infection. So, we considered that the possibility of re-infection was relatively low. However, we think your opinion is very reasonable, and we had re-writen the discussion part, which put the first reason of re-positive results as residual viral loads.

**Answers to Review 1**

**1.A research from South Korea reported 292 re-positive COVID 19 cases, they determined the re-positive is due to the limits of test method, and the remnants of the virus was picked up in the test. What do you think about it?**

Reply：Thank you very much for this question. Your two comments on this article are very important. We had been thinking about the two questions you raised.

We had read the article you mentioned. It is a brief report based on the data given by South Korea Centers for Disease Control and Prevention. Most of the information mentioned in the article is consistent with the results of our article, such as most of the re-positive patients showed mild symptoms and were concentrated in relatively young age groups.

In fact, we were also very interested in what causes the re-positive results in recovered patients. Regrettably, it was strictly limited in management of covid-19 patients’ specimens here. We could not conduct specific studies on that by ourselves and can only learn from other researchers’ article. According to the current results，we believed that the author’s description of dead virus or virus fragments in re-positive patients was a highly likely. However, to confirm what caused this phenomenon in re-positive patients requires further research. We will continue to monitor related article.

2. **Another research in Wuhan claim that the positive rate of virus with pharyngeal swabs in COVID 19 patients is only 32% (126/398). Viral load changes as the disease progresses, false negative result at discharge may affect the ratio of RPR in COVID 19 patients.**

Reply：The question you raised is very important.

In the progression of covid-19 patients’ infection, their viral load had experienced a rapid increase in the early stage of the disease to a slow decrease in the recovery period. Pharyngeal swabs were not the best choice from the perspective of detection accuracy. But pharyngeal swabs had the advantages of convenient collection, it was the main method for specimen collection in the early stage. In order to maximize the reliability of results, we required that the nucleic acid collectors in each ward be a fixed number of experienced medical staff in Huangshi's covid-19 management. At the same time, we required that each patient underwent two independent nucleic acid tests before being discharged from the hospital, with an interval of more than 24 hours. Through these two methods, we tried our best to improve the accuracy of the nucleic acid results in the research. And thanks to your advice, we re-discussed the issue in our manuscript to make readers be aware of the possibility of false negative result.

**Answers to Review 2:**

1. **The introduction is very brief, it would be nice if authors can add more about SARS-CoV-2 in it.**

Reply：Thank you for your suggestion. We had added introduction of SARS-CoV-2 in article in revised manuscript.

2. **Why only 383 patients were chosen for the study?**

Reply：The number of patients we included in the study was not actually set by us. After we determined the deadline for follow-up, we screened all patients from the two participating units before the deadline. All eligible patients have been included in the study. In terms of proportion, 84.4% of the patients discharged from hospital before March 1st in Huangshi City have been included in this study, which is a good representative.

3. **Line 86- If the patients were tested positive during the discharge period, why they kept in quarantine instead of continuing with the same treatment? Please explain.**

Reply**：**We are sorry that our description in the article is not clear enough. All discharged patients will be quarantined for 14 days. After the quarantine period, everyone would undergo nucleic acid testing. Negative patients could go home, and positive patients will be admitted to the hospital to continue treatment. In the revised manuscript, we described this process more meticulously.

4. **What were the parameters taken while data collection as authors mentioned a data collection form, it would be beneficial for readers if authors can provide the sample form in the supplementary.**

Reply：We really appreciate your suggestion. In the revised manuscript, we provided sample form in supplementary file.

5. **Among the comorbidities, most of the focus is on hypertension and chronic diseases in respiratory system; while as it would have been interesting to see RPR in the immunocompromised such as HIV positive patients. Authors have completely ignored this factor; however, it will be an interesting inclusion.**

Reply：Thank you very much for the question. It is really important. We are also very interested in the re-positive performance of patients with abnormal immune status. In our study, all the patients when they admitted to hospital were inquired of detailed personal history, including HIV infection. Due to the limited conditions, not all the patients were screened for HIV test during hospitalization. According to your advice, we screened the data again and we found that none of the patients had a history of AIDS. Among all the patients, 63 of them underwent HIV antibody test due to possible blood transfusion, and all of their results were negative. Then we searched the literatures. The epidemiological study from Huangshi Center for Disease Control and Prevention showed that there were 1 518 HIV/AIDS cases reported from 2011 to 2017 in Huangshi city [4], while the total population of Huangshi is 2.69 million. The infection rate of HIV in Huangshi is relatively low. That could be the reason why HIV patients were rare in our study.

**References:**

1. Gudbjartsson DF, Norddahl GL, Melsted P, Gunnarsdottir K, Holm H, Eythorsson E, et al. Humoral Immune Response to SARS-CoV-2 in Iceland. N Engl J Med. United States; 2020;

2. Deng W, Bao L, Liu J, Xiao C, Liu J, Xue J, et al. Primary exposure to SARS-CoV-2 protects against reinfection in rhesus macaques. Science. 2020;369:818–23.

3. Kang H, Wang Y, Tong Z, Liu X. Retest positive for SARS-CoV-2 RNA of “recovered” patients with COVID-19: Persistence, sampling issues, or re-infection? J Med Virol. 2020;

4. Fu Xiong, Ruiqing Xie, Xianzhou Ke ZQ. AIDS epidemic and epidemic characteristics in Huangshi, 2011- 2017. China Trop Med [Internet]. China Tropical Medicine; 2018;18:906. Available from: http://www.cntropmed.com/CN/abstract/article_13504.shtml
