# Supplementary material for: Clinical characteristics of re-positive COVID-19 patients in Huangshi, China: A retrospective cohort study
Source: PLoS One. 2020 Nov 4;15(11):e0241896. doi: 10.1371/journal.pone.0241896 (PMC7641455; doi:10.1371/journal.pone.0241896)
Supplement: Attachment — Submitted filename: Response to reviewers.docx [file pone.0241896.s008.docx]

**Dear editor:**

Thanks a lot for having reviewed our manuscript.   Now we have revised the manuscript according to the reviewers’ comments. Most of the revisions are in the manuscript. Some explanations regarding the revisions of our manuscript are as follows. Just as we communicated in the E-mail on Oct 15, comments of 3 and 6 from the reviewer # 3 were ignored here.

**Answers to Review #3**

1. In this study. 383 patients in Huangshi hospital of Traditional Chinese Medicine (TCM) and Huangshi hospital of Youse was included, but the Ethics Statement showed that the study was approved only by the Research Ethics Commission of Huangshi Hospital of Traditional Chinese Medicine. It should has another Ethics Statement from Huangshi hospital of Youse. Please provide the Ethics statement to address this issue.

Reply：Thank you very much for reminding. In fact, this study was approved by the ethics committees of two hospitals at the beginning of the study. We have modified the inaccurate description in the manuscript and provided the ethics committee approval documents issued by these two hospitals (form Line 103 to Line 104).

2. In this study, all patients were diagnosed with COVID-19 according to the Chinese management guideline for COVID-19 (version 6.0). This guideline was published in the Feb. 18th 2020, how could all patients be diagnosed according to this guideline? Some patients must be diagnosed with covid-19 before the guideline being published.

Reply：Our description here is not rigorous. We had made corrections in the manuscript (On Line 83 and Line 101).

3. Two patients, whose cough and chest tightness worsened during quarantine after they were discharged, were considered as non-recovering patients who were not included in our study. But why they were considered as non-recovering patients, not the re-positive recovering (RPR) patients? They were negative when they were discharged from the hospital.

Reply：This issue had been corrected in revised manuscript. Just as we communicated in the E-mail on Oct 15, this comment was ignored here.

4. About 30·68% of them had comorbidities. The most common comorbidities were hypertension (n=72，18·8%) followed by diabetes (n=33,8·62%) and coronary heart disease(n=16, 4·18%). How many patients had chronic disease? And how many patients who had two comorbidities at least For example patients had hypertension and diabetes together.

Reply：Your comments are very valuable. We had added a description about this in Table 1and manuscript (From Line 170 to Line 172, Line 234 to Line 235).

5. Multivariate analysis showed that the arbidol reduced the probability of repositive outcome. But it did`t give the criteria for the use of drugs. Who had received the arbidol and who hadn't?

Reply：This question is very good. However, at the beginning of the year, in response to the unprepared attack of COVID-19, we did not have an accurate understanding of the therapeutic value of Abidol, or whether it was more effective in certain groups of people. You could find that our guideline has been updated from the 2st edition to the 6th edition within a few months, and our understanding of covid-19 has been improving and changing until now. Therefore, in this article, we have not stipulated what kind of patients should or should not use Arbidol. Each doctor in charge chose combination of drugs based on his or her professional knowledge.

6. In Multivariate analysis, there showed “a younger age (adjusted HR 0·075, 95% CI 0·009-0·657; P =0·0193),” “the arbidol reduced the probability of repositive outcome (adjusted HR 0·048, 95% CI:0·010-0·231; P =0·0002).”, but in DISCUSSION, “There was no difference in demographics, epidemiological features and treatment between the RPR and non-RPR groups by univariate analysis.” Please clarify.

Reply：This issue had been corrected in revised manuscript. Just as we communicated in the E-mail on Oct 15, this comment was ignored here.
